# ATTR amyloidosis during the COVID-19 pandemic: insights from a global medical roundtable

**DOI:** 10.1186/s13023-021-01834-0

**Published:** 2021-05-06

**Authors:** Thomas H. Brannagan, Michaela Auer-Grumbach, John L. Berk, Chiara Briani, Vera Bril, Teresa Coelho, Thibaud Damy, Angela Dispenzieri, Brian M. Drachman, Nowell Fine, Hanna K. Gaggin, Morie Gertz, Julian D. Gillmore, Esther Gonzalez, Mazen Hanna, David R. Hurwitz, Sami L. Khella, Mathew S. Maurer, Jose Nativi-Nicolau, Kemi Olugemo, Luis F. Quintana, Andrew M. Rosen, Hartmut H. Schmidt, Jacqueline Shehata, Marcia Waddington-Cruz, Carol Whelan, Frederick L. Ruberg

**Affiliations:** 1 Department of Neurology, Columbia University, New York, NY USA; 2 Vienna General Hospital, Vienna, Austria; 3 Boston University, Boston, MA USA; 4 University of Padova, Padova, Italy; 5 University Health Network, Toronto, ON Canada; 6 Centro Hospitalar Universitário do Porto, Porto, Portugal; 7 Referral Center for Cardiac Amyloidosis, Cardiology Department, APHP-Henri Mondor Hospital, Creteil, France; 8 Mayo Clinic, Rochester, MN USA; 9 Penn Presbyterian Medical Center, Philadelphia, PA USA; 10 University of Calgary, Calgary, AB Canada; 11 Massachusetts General Hospital, Boston, MA USA; 12 National Amyloidosis Centre, Royal Free Hospital, London, UK; 13 Hospital Puerta de Hierro of Madrid, Madrid, Spain; 14 Cleveland Clinic, Cleveland, OH USA; 15 Akcea Therapeutics, Boston, MA USA; 16 University of Pennsylvania, Philadelphia, PA USA; 17 Columbia University, New York, NY USA; 18 University of Utah, Salt Lake City, UT USA; 19 Hospital Clinic, University of Barcelona, Barcelona, Spain; 20 University Hospital of Münster, Münster, Germany; 21 Federal University of Rio de Janeiro, Rio de Janeiro, Brazil; 22 Section of Cardiovascular Medicine, Department of Medicine and Amyloidosis Center, Boston University School of Medicine, Boston Medical Center, Boston, MA USA

**Keywords:** COVID-19, SARS-CoV-2, Amyloidosis, Rare disease, ATTR

## Abstract

**Background:**

The global spread of severe acute respiratory syndrome coronavirus 2 (SARS-CoV-2) infection causing the ongoing coronavirus disease 2019 (COVID-19) pandemic has raised serious concern for patients with chronic disease. A correlation has been identified between the severity of COVID-19 and a patient’s preexisting comorbidities. Although COVID-19 primarily involves the respiratory system, dysfunction in multiple organ systems is common, particularly in the cardiovascular, gastrointestinal, immune, renal, and nervous systems. Patients with amyloid transthyretin (ATTR) amyloidosis represent a population particularly vulnerable to COVID-19 morbidity due to the multisystem nature of ATTR amyloidosis.

**Main body:**

ATTR amyloidosis is a clinically heterogeneous progressive disease, resulting from the accumulation of amyloid fibrils in various organs and tissues. Amyloid deposition causes multisystem clinical manifestations, including cardiomyopathy and polyneuropathy, along with gastrointestinal symptoms and renal dysfunction. Given the potential for exacerbation of organ dysfunction, physicians note possible unique challenges in the management of patients with ATTR amyloidosis who develop multiorgan complications from COVID-19. While the interplay between COVID-19 and ATTR amyloidosis is still being evaluated, physicians should consider that the heightened susceptibility of patients with ATTR amyloidosis to multiorgan complications might increase their risk for poor outcomes with COVID-19.

**Conclusion:**

Patients with ATTR amyloidosis are suspected to have a higher risk of morbidity and mortality due to age and underlying ATTR amyloidosis-related organ dysfunction. While further research is needed to characterize this risk and management implications, ATTR amyloidosis patients might require specialized management if they develop COVID-19. The risks of delaying diagnosis or interrupting treatment for patients with ATTR amyloidosis should be balanced with the risk of exposure in the health care setting. Both physicians and patients must adapt to a new construct for care during and possibly after the pandemic to ensure optimal health for patients with ATTR amyloidosis, minimizing treatment interruptions.

## Background

The pandemic associated with the coronavirus disease 2019 (COVID-19) syndrome, which is caused by severe acute respiratory syndrome coronavirus 2 (SARS-CoV-2) infection, has rapidly become a serious threat to global public health. The impact of SARS-CoV-2 infection on patients with most chronic conditions is largely unknown. Studies assessing characteristics associated with poor outcomes after SARS-CoV-2 infection have identified a correlation between the severity of COVID-19 and a patient’s preexisting comorbidities (eg, diabetes mellitus, obesity, hypertension, cardiovascular disease, cerebrovascular disease, chronic obstructive lung disease, chronic liver disease, chronic kidney disease) [1–4]. Risk factors for COVID-19 complications also include age (eg, > 65 years), male sex, race, hypertension, diabetes and increased body mass index (BMI) [3, 4]. Furthermore, patients with chronic conditions often require stable access to healthcare for diagnostic testing, treatment, and routine monitoring. The pandemic has significantly impacted care for chronic disease by causing a shift in healthcare resources to manage acutely ill patients with COVID-19 with subsequent implementation of telemedicine for many other cases. This has led to restrictions on diagnostic testing for many chronic diseases due to temporary closures of clinics and hospitals.

Extensive literature on the subject has indicated that SARS-CoV-2 initially infects the respiratory system, leading to a constellation of symptoms that may include dyspnea, hypoxemia, loss of taste/smell, sore throat, dry cough, headache, and fever along with fatigue, muscle pain and gastrointestinal symptoms such as diarrhea, vomiting and abdominal pain (Table 1) [1, 3–13]. Severely affected patients may develop pneumonia, acute respiratory distress syndrome (ARDS), thromboembolic complications including pulmonary embolism, myocardial injury including myocarditis and/or heart failure, kidney failure, septic shock and/or multi-system organ failure, and death (Table 1) [1, 5, 7, 8, 10, 12, 14, 15]. Organ damage is thought to be mediated via a severe immune activation, or cytokine storm, and an endotheliopathy that induces microcirculatory dysfunction, leading to many of the clinical sequelae including thromboembolic complications and microangiopathy [15, 16].

**Table 1:** Comparison of the clinical manifestations of ATTR and COVID-19

	ATTR	COVID-19	References
**Cardiovascular**	**HF**, rhythm disturbances, conduction block, **dyspnea**, syncope, **palpitations**, **elevated biomarkers, thromboembolism**	**HF**, **myocardial injury**, myocarditis, acute coronary syndromes, **palpitations**, **dyspnea**, **elevated biomarkers, thromboembolism**	[11, 14, 17, 26, 30, 63, 64, 66]
**Gastrointestinal**	**Diarrhea**, constipation, **loss of appetite**, **nausea**	**Diarrhea**, **loss of appetite**, **nausea**/vomiting, gastroenteritis	[6, 9, 26, 29, 91, 92]
**Hematologic and Immunologic**	Increased pro-inflammatory cytokines, increased propensity to left atrial thrombosis	Cytokine storm, venous thrombosis, lymphopenia, venous/arterial thromboembolism, coagulopathy, microangiopathy	[15, 16, 20–22, 40, 56]
**Musculoskeletal**	CTS, spinal stenosis, trigger finger, shoulder, knee and hip surgery	Arthralgia, myalgias	[11, 31, 81, 103, 104]
**Renal**	Proteinuria, renal failure	AKI, ATN	[26, 30, 40, 48, 93, 94]
**Neurologic**	CNS: **headache**, ataxia**, seizures**, **stroke-like episodes**	**Headache**, dizziness, **seizures**, mental status changes, muscle pain, **stroke,** neuropathy (eg, AIDP), **myopathy,** myelitis, anosmia, hypogeusia, dysgeusia	[12, 26, 30, 32, 34, 70–72, 75–77, 105, 106]
	Peripheral neuropathy: numbness/tingling in hands and feet, neuropathic pain, walking disability, loss of balance		
	Autonomic neuropathy: urinary retention and incontinence; erectile dysfunction; orthostatic hypotension; gastrointestinal manifestations		
**Pulmonary**	Impairment from alveolar-septal amyloidosis	Cough, shortness of breath, pneumonia, ARDS	[1, 4, 5, 44, 45, 47, 48]
	Pulmonary hypertension due to left-sided heart disease		
**Other/Constitutional**	Unintentional weight loss	Fever, fatigue	[5, 7, 26]

Bold text indicates overlapping manifestations or symptoms when comparing ATTR and COVID-19. Cardiac and musculoskeletal manifestations are most predominant in patients with ATTRwt amyloidosis; clinical manifestations in peripheral and autonomic nerves, along with the kidney, and eye are rarer. *AIDP* Acute Inflammatory Demyelinating Polyneuropathy, *AKI* Acute kidney injury, *ARDS* Acute respiratory distress syndrome, *ATN* Acute tubular necrosis, *ATTR* Amyloid transthyretin, *CNS* Central nervous system, *COVID-19* Coronavirus disease 2019, *CTS* Carpal tunnel syndrome, *HF* Heart failure

**Fig. 1:**
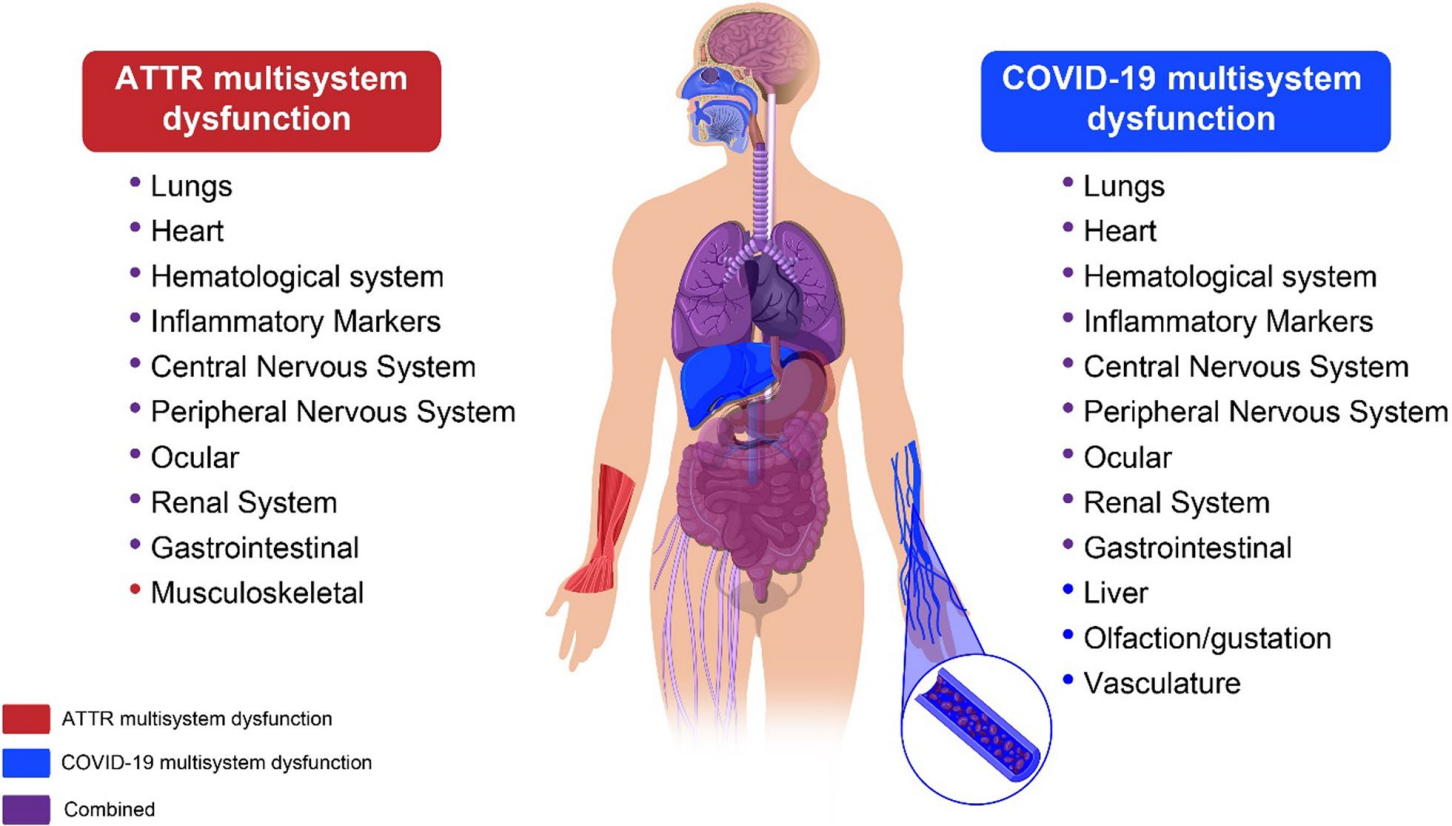
ATTR amyloidosis and COVID-19 have overlapping clinical features including common underlying cardiovascular, gastrointestinal, and hematologic symptoms. *ATTR* Amyloid transthyretin

Patients with amyloid transthyretin (ATTR) amyloidosis represent a population that is likely to be particularly vulnerable to COVID-19 due to the multisystem nature of the disease (Fig. 1), and in many cases, the advanced age of the patients along with presensitization of their immune system due to the chronic inflammation that is a byproduct of abnormal amyloid deposits [17–23]. While substantiating data are not available, the authors strongly suspect that patients with ATTR amyloidosis are at increased risk of poor clinical outcomes with COVID-19 [24]. There are two types of ATTR amyloidosis: wild-type (ATTRwt) and hereditary (ATTRv [variant]). ATTRwt amyloidosis is caused by the misfolding into amyloid fibrils of the native, genetically normal, transthyretin (TTR) protein, whereas ATTRv amyloidosis is caused by misfolding and fibril aggregation due to destabilization of the protein tetramer that is caused by a single amino acid substitution in the *TTR* gene. Regardless of origin, both forms of ATTR amyloidosis result in aggregation of TTR protein into fibrils that deposit into various tissues and organs, resulting in multisystem dysfunction [25–28], but the mutation-originated folding error may cause a more clinically severe or earlier onset of disease in certain cases. Amyloid deposition causes clinical manifestations in multiple organ systems including restrictive cardiomyopathy, progressive motor and sensory polyneuropathy, autonomic neuropathy, and musculoskeletal manifestations along with gastrointestinal, ocular, and renal disturbances (Table 1) [26, 29–31]. The clinical presentation of ATTR amyloidosis depends on the type. ATTRwt amyloidosis primarily causes cardiac dysfunction and is seen predominantly in males, although patients can less commonly present with manifestations in the peripheral and autonomic nerves (Table 1) [29, 30]. Most patients with ATTRv amyloidosis experience a mixed phenotype with both cardiac and neuropathic symptoms; however, depending on the TTR mutation, patients may experience predominantly cardiac or neuropathic symptoms (Table 1) [29, 30, 32–34]. Both types of ATTR amyloidosis can present with musculoskeletal manifestations, including carpal tunnel syndrome, spinal stenosis, and other orthopedic manifestations (Table 1) [31]. Patients with ATTR amyloidosis are more often male [30, 35, 36] and the age of disease onset can vary. Clinical presentation of ATTRwt amyloidosis typically occurs in patients 50 years or older, whereas ATTRv amyloidosis can be classified as early or late onset based on the presentation of symptoms before or after the age of 50, respectively [17–19, 37–39].

It is challenging to predict the impact of SARS-CoV-2 infection on patients with ATTR amyloidosis and no data yet exist to guide treatment decisions; however, given pre-existing multisystem involvement, patients with ATTR amyloidosis are likely at risk for developing severe COVID-19 [24]. To explore the impact of COVID-19 on the health and care of patients with ATTR amyloidosis, a global expert panel that included 25 physicians from 10 different countries who specialize in cardiology, neurology, hematology, infectious disease, nephrology, pulmonology, and gastroenterology, met virtually on May 11–12, 2020. The aim of this position paper is to share with the broader medical community the perspectives communicated during this global medical roundtable (with relevant updates provided via email exchange communication in January 2021) regarding the system-specific susceptibility of patients with ATTR amyloidosis to COVID-19, as well as how the multisystem nature of ATTR amyloidosis might compound the risks of morbidity and mortality of COVID-19. This article will summarize discussions pertaining to specific treatment considerations, along with physician experiences related to patient diagnosis, access to treatment, disruption of referral networks and workarounds, and impact of the pandemic on ongoing clinical research.

## Main text

### Specialty-specific perspectives on the anticipated impact of COVID-19 in patients with ATTR amyloidosis

Given the multisystem clinical manifestations of ATTR amyloidosis, specialty-specific challenges are anticipated for physicians managing patients with ATTR amyloidosis who develop multiorgan complications from COVID-19. Several articles have separately summarized what is known about the clinical manifestations of ATTR amyloidosis [26, 40] and COVID-19 [7, 11, 41–43]. Patients with ATTR amyloidosis may have clinical manifestations that vary depending on the pattern of amyloid deposition and the severity of organ dysfunction. Many of the involved organs systems for ATTR amyloidosis and COVID-19 are overlapping (eg, cardiac, gastrointestinal, renal), whereas other manifestations, although unique, may impact therapeutic decision-making (Fig. 1, Table 1). The following sections provide an in-depth overview of each organ system and the potential impact of COVID-19 on ATTR amyloidosis.

#### Pulmonary

The respiratory distress caused by COVID-19 will likely have a severe impact on patients with ATTR amyloidosis, as amyloid fibril infiltration can be found throughout the respiratory tract at autopsy, and patients with cardiac involvement of ATTR (ATTR-CM) may have pre-existing chronic elevation of pulmonary venous pressure. ATTR amyloidosis can rarely induce pulmonary disease by (a) infiltration of the alveolar-septal space or (b) pulmonary hypertension resulting from chronically elevated heart filling pressures. Although rare, cases of alveolar-septal amyloidosis attributed to TTR amyloid infiltration of the alveolar septa and vessel walls have been described [44, 45]. Pulmonary impairment due to alveolar-septal amyloidosis is rarely the dominant clinical manifestation and, as a result, it is mostly diagnosed post mortem [45, 46]. Alveolar-septal amyloidosis has been identified in patients with ATTRwt and ATTRv amyloidosis [44, 45, 47]. Alternatively, heart failure in ATTR amyloidosis is common, resulting in pulmonary edema and elevated post-capillary pulmonary hypertension.

Clinical features associated with COVID-19 range from mild respiratory illness to severe acute respiratory disease [5]. Patients with COVID-19 frequently develop pneumonia—characterized by fever, cough, and dyspnea along with bilateral infiltrates observed by chest imaging [5]. Early stages of infection are characterized by flu-like symptoms, and patients may subsequently develop viral pneumonia characterized by pulmonary inflammation and coagulopathy, requiring hospitalization [48]. Late stages of infection are characterized by fibrosis with impaired gas exchange [48]. The mechanisms underlying the pathophysiology behind these COVID-19–related respiratory phenotypes are poorly understood.

Recent reports have described cases of patients infected with COVID-19 whose respiratory status appears stable despite significant hypoxemia [49–51]. Physicians should be aware that patients with ATTR amyloidosis (multi-organ disease) and COVID-19 are unlikely to tolerate hypoxemia, as pre-existing heart and kidney involvement may magnify the organ injuring effects of hypoxia. Physicians should consider recommending use of a pulse oximeter or an oximeter app as an at-home monitoring device that, if combined with persisting symptoms, could alert a patient to seek medical attention.

#### Hematologic and immunologic

The dramatic cytokine storm observed with COVID-19 will likely have an impact on patients with a multisystem disease, such as ATTR amyloidosis, in which proinflammatory cytokines might be elevated at baseline. An immune system weakened because of age, chronic illness, or malnourishment, as is often seen in patients with ATTRv amyloidosis, might increase the risk for poor outcomes after SARS-CoV-2 infection [15, 23]. Recent studies suggest that ATTR amyloidosis is also a disease with an inflammatory component, finding increased levels of pro-inflammatory cytokines in the serum of patients with ATTRv amyloidosis compared to healthy controls [20–23]. However, the clinical significance of inflammation in patients with ATTR amyloidosis has not yet been fully clarified.

Inflammatory cytokine levels are also increased in patients with COVID-19. A subset of patients with COVID-19 develop severe immune activation (cytokine storm) in response to SARS-CoV-2 infection, resulting in systemic inflammatory response syndrome (SIRS)-like symptoms, acute respiratory distress syndrome (ARDS), and multi-system organ failure [16]. This is characterized by a fulminant increase in cytokine and chemokine levels including IL-2, IL-6, IL-7, IL-10, GCSF, IFN- γ, and TNF-α in patients with severe COVID-19 [5, 16, 48]. These observations have prompted the exploration of immune modulators, such as the IL-6 antagonist tocilizumab or IL-1 antagonist anakinra, as potential treatments for severe COVID-19. Physicians have noted that patients with COVID-19 who died from the infection initially developed ARDS with hyperpermeability of the alveoli. The cytokine storm induced by COVID-19 is suspected to be the underlying cause inducing alveolar epithelial damage [16]. In addition, alveolar hemorrhage might be triggered by endothelial injury associated with cytokine release due to SARS-CoV-2 infection, causing bleeding into the lungs. The experts on the current panel speculated that the underlying mechanism is likely more complicated than simply an antiviral response, with significant alveolar damage likely contributing to noncardiogenic pulmonary edema, leading to severe respiratory failure and hypoxia. For this reason, nonspecific anti-inflammatory agents such as corticosteroids (dexamethasone) have proven among the few effective agents in reducing the clinical severity of COVID-19 [52].

Coagulopathy has been observed in patients with COVID-19 and is presently appreciated as a fundamental derangement that presages severe complications. The development of coagulation test abnormalities is most likely a result of the profound inflammatory response observed in these patients. This “COVID-19-associated coagulopathy” in early infection reflects abnormalities in coagulation tests (initially high D-dimer and fibrin/fibrinogen-degradation products and later high activated partial thromboplastin time (aPTT) and prothrombin (PT) [3, 15, 48]), but does not fulfill the usual definition of a clinical coagulopathy in which there is bleeding. In contrast, patients are prone to thrombosis, which is a function of their hypercoagulopathy and their microangiopathy. In addition, pernio-like lesions (also known as chilblains or “COVID-toes”), characterized by red or purple skin lesions on the hands and/or feet, are observed in patients with COVID-19 and can be manifestations of distal thrombosis/embolism or retiform purpura [53–55]. Such observations have prompted a more aggressive approach toward systemic thromboprophylaxis or full anticoagulation in different scenarios.

Physicians should be mindful that patients with ATTR-CM are at an increased risk of intra-cardiac thrombus and embolization, which could be exacerbated by the pro-thrombotic state observed with COVID-19 [15, 40, 56]. Moreover, an immune system weakened by age, chronic illness, or malnourishment, as is often seen in patients with ATTR amyloidosis, might add risk for poor outcomes because of SARS-CoV-2 infection in this patient population [15, 23]. Data on the clinical presentation of COVID-19 in patients with solid organ transplantation remains limited [57]. Management of COVID-19 with immunosuppression needs to be carefully considered for patients with ATTR amyloidosis and a history of liver transplantation [58–60]. Physicians should consider using standard laboratory tests (eg, D-dimer, fibrinogen, ferritin, troponins, B-type natriuretic peptide [BNP] or N-terminal (NT)-pro hormone -BNP [NT-pro-BNP]) to guide therapy, particularly in patients with ATTR amyloidosis who might already have baseline values for these tests.

#### Cardiovascular

Patients with pre-existing cardiovascular disease and hypertension have a heightened vulnerability to more severe disease with worse clinical outcomes if they develop COVID-19 [14, 61, 62]. The presence of heart failure along with the advanced age of patients with ATTR-CM, particularly ATTRwt amyloidosis, is suspected to increase the potential risk for worse outcomes if these patients develop COVID-19. Cardiovascular manifestations of ATTR amyloidosis are characterized by an increase in left ventricular (LV) mass, and clinical heart failure progressing to restrictive physiology and the presence of rhythm disturbances, conduction system abnormalities, dyspnea, syncope, and palpitations [17, 26, 30, 63, 64]. Heart failure progressively worsens over time, with patients with ATTR amyloidosis experiencing progressive diastolic dysfunction, elevated biomarkers (eg, troponin I, BNP or NT-proBNP), rapid decline in functional capacity, and a decrease in LV ejection fraction (LV-EF) [17]. More-advanced ATTR amyloidosis can also result in heart failure with midrange or reduced ejection fraction [64, 65]. Other cardiovascular symptoms of ATTR amyloidosis may include orthostatic hypotension due to autonomic dysfunction [26, 30, 32].

Cardiovascular manifestations of COVID-19 include clinical signs of myocardial ischemia or injury, arrhythmias, myocarditis, electrocardiogram (ECG)-abnormalities mimicking ST-segment elevation myocardial infarction, and heart failure [11, 14, 66]. Right ventricular dysfunction is common (25–50%) in the setting of ARDS due to COVID-19 [14, 67]. Elevated biomarkers, such as cardiac troponin I or T and BNP or NT-proBNP in COVID-19 patients are associated with adverse outcomes, including intensive care unit (ICU) admission and mortality [3, 14]. These markers are also increased in ATTR-CM, thereby potentially confounding the assessment of the clinical impact of COVID-19. The pathogenesis of myocardial inflammation associated with COVID-19 may be owing to direct viral involvement of the myocardium mediated by angiotensin converting enzyme 2 (ACE2) receptor [11, 66], in addition to the cardiac effects of a cytokine storm or related to coronary microvasculature thrombosis. Cardiovascular emergencies related to COVID-19 include acute coronary syndromes (ACS) that includes ST-elevation myocardial infarction (STEMI) and non-ST-elevation acute coronary syndromes (NSTE-ACS), most typically with coronary angiography demonstrating no obstructive lesions [68]. Circulatory collapse from profound systolic dysfunction resulting from myocarditis is also a dreaded complication of COVID-19. Orthostatic hypotension from ATTR amyloidosis may be exacerbated with acute COVID-19 owing to cytokine increase and distributive hemodynamics.

All panel experts agreed that any patient with limited cardiac reserve in response to stress will likely be at higher risk of death from SARS-CoV-2 infection, particularly in combination with elevated pulmonary vascular resistance associated with hypoxemia. The overall lack of cardiac reserve in a patient with ATTR amyloidosis and restrictive cardiomyopathy may result in rapid clinical deterioration. Poor outcomes are more likely in patients who develop left-sided heart failure, which is typical of ATTR-CM, or right-sided heart failure, which is commonly observed with respiratory distress syndrome due to COVID-19, in combination with severe lung inflammation that affects heart function. Such patients have stiff, non-compliant ventricles that operate with a narrower window of tolerance for perturbations in volume status. The tremendous systemic stress caused by COVID-19—tachycardia and heart rhythm disturbances, hypoxemia, hypotension—will likely be poorly tolerated by an amyloid-affected heart.

Patients with ATTR-CM or ATTRv amyloidosis due to the p.V142I (ATTRV122I) *TTR* mutation or other *TTR* variant cardiomyopathies will likely be significantly affected by COVID-19, perhaps disproportionately so. Physicians should consider whether the threshold to admit patients with ATTRV122I or ATTR amyloidosis with cardiac involvement should be different because of the potentially elevated risk for severe disease related to COVID-19. Furthermore, panel experts endorsed consideration of thromboprophylaxis in patients with ATTR-CM and COVID-19 given the propensity for intracardiac thrombosis and prothrombotic state elicited by infection, per recent specialty society recommendations [69].

#### Neurologic

Neurological disorders that may develop with COVID-19 are likely to worsen the clinical condition of patients with ATTR amyloidosis. Polyneuropathy associated with ATTR amyloidosis (ATTR-PN) is characterized by symmetrical length-dependent peripheral neuropathy [26, 34]. Heterogeneous clinical manifestations reflective of the pattern of progression and impacted nerve fiber class at each stage are experienced by patients with ATTR-PN [34, 70–72]. Early symptoms include burning pain, with older patients also experiencing numbness and loss of pain and temperature sensation; despite these, the ability to perceive touch pressure and joint position is relatively preserved [26, 70]. With disease progression, patients increasingly experience progressive lower limb numbness, weakness, and gait imbalance [30, 72–74].

Patients with COVID-19, particularly those with severe disease, experience neurologic manifestations with symptoms such as dizziness, headache, seizures, impaired consciousness, and acute cerebrovascular disease [12, 75, 76]. Central nervous system (CNS) complications are reported to manifest early in the course of infection [12]. Panel physicians have observed an elevated risk for intracranial hemorrhage in critically ill patients with COVID-19 supported by extracorporeal membrane oxygenation (ECMO). Along with significant peripheral venous and arterial thrombosis, both venous and arterial, stroke has also been observed [12, 77]. Panel experts had mixed experiences with stroke occurrence due to COVID-19. Although some physicians have not seen an increase, it was noted that stroke in some patients with COVID-19 seemed to occur later in the disease course after the initial presentation [77]. In Italy, physicians have observed a decrease in cases of minor strokes, transient ischemic attacks (TIA), and transfers for strokes along with longer onset-to-door and door-to-treatment times for major strokes [78], likely due to patient reluctance to go to the hospital, especially for mild strokes. Physicians should be aware that the risk of ischemic stroke is high among critically ill elderly patients with vascular risk factors similar to those in patients with cardiac manifestations of ATTR amyloidosis [12, 64].

Reports of the effect of COVID-19 on the peripheral nervous system (PNS) and muscle are more limited and it is unclear whether there is an increase in the incidence of acute demyelinating polyneuropathy (AIDP [eg, Guillain–Barré syndrome]) and flaccid myelitis [12, 75, 79–82]. Physicians report mixed neurologic manifestations in their patients. Physicians in Latin America and Canada (Toronto) have not observed an increase, whereas in Italy, physicians have observed an increase in the incidence in Guillain–Barré syndrome after COVID-19 [83]. A large population study in England did not see an increase in Guillain–Barré syndrome associated with COVID-19 [84]. Mononeuritis multiplex has also been seen in patients with COVID-19 [85, 86]. It is unknown whether the neurologic manifestations reported in patients with COVID-19 are a direct result of the neuroinvasive properties of the virus or a consequence of cardiorespiratory failure and multisystem dysfunction [75, 81]. Systematic reviews of the literature suggest that COVID-19–related neurological manifestations need to be better characterized. In addition, patients with prolonged ICU hospitalization frequently develop critical-illness–related myopathy [87–89]. A growing number of reports describe an association of COVID-19 and critical-illness related myopathy and neuropathy, characterized by mainly proximal muscle wasting and, less often, peripheral neuropathy in the lower and upper limbs [75, 90].

Neurologists at this roundtable had not observed worsening polyneuropathy in patients with ATTR amyloidosis during the COVID-19 pandemic; however, many of their patients had not yet been diagnosed with COVID-19. Patients with ATTR amyloidosis and COVID-19 who developed a critical-illness–related neuropathy might be mistaken to have progression of ATTR-PN. This is less likely to occur with COVID-19–associated Guillain–Barré syndrome, which has a different time course, pattern of involvement, and neurophysiology. If a patient with ATTR amyloidosis exhibits rapidly progressive polyneuropathy is it likely because of viral nerve injury, as polyneuropathy associated with ATTR amyloidosis does not accelerate in that fashion. It is unclear whether patients with ATTR-PN will be more susceptible to COVID-19–related inflammatory autoimmune reactions in the PNS. Overall, if a patient with ATTR-PN experiences a second type of neuropathy, the patient’s clinical condition will likely be worse.

#### Gastrointestinal

Clinical manifestations of gastrointestinal involvement due to COVID-19 may confound the underlying gastrointestinal symptoms experienced by patients with ATTR amyloidosis. In patients with ATTR amyloidosis, autonomic dysfunction can also manifest as gastrointestinal symptoms such as diarrhea and constipation, nausea, delayed gastric emptying, vomiting, early satiety, and incontinence [26, 29, 91]. The prevalence of gastrointestinal symptoms in patients with ATTR amyloidosis significantly increases over time and is associated with devastating complications, negatively impacting the patient’s nutritional status and quality of life [91].

Patients with COVID-19 have reported gastrointestinal symptoms including diarrhea, nausea and/or vomiting, abdominal pain and loss of appetite [6, 9, 92]. Furthermore, viral gastroenteritis, characterized by the presence of white blood cells in stool samples and a positive occult blood test, has been reported in a subset of patients with COVID-19 [6]. Studies have described patients with COVID-19 reporting gastrointestinal symptoms days prior to the development of respiratory symptoms or fever, and in some cases, patients experience only digestive symptoms [6, 9]. SARS-CoV-2 RNA has been detected in stool samples, even in patients without gastrointestinal symptoms and sometimes after viral RNA is undetectable in the respiratory tract [6]; however, it is unknown whether fecal–oral transmission of SARS-CoV-2 infection occurs.

Given the similarity in gastrointestinal symptoms, it might be challenging for patients to distinguish between those manifestations related to ATTR amyloidosis and those due to COVID-19, particularly in the presence of diarrhea and loss of appetite. Although there is no data describing the impact of SARS-CoV-2 infection in patients with ATTR amyloidosis, due to the known gastrointestinal manifestations of ATTR amyloidosis, particularly ATTRv amyloidosis, current roundtable expert physicians speculated that patients with ATTR amyloidosis are more vulnerable to developing the gastrointestinal symptoms associated with COVID-19.

#### Renal

The interplay of ATTR amyloidosis and COVID-19 may cause an increased risk for renal dysfunction that could impact patient management. Although rare, TTR amyloid deposits in the kidney can result in renal manifestations including albuminuria, azotemia, and renal dysfunction, which along with comorbidities such as heart failure can increase the risk of renal dysfunction in patients with ATTR amyloidosis [26, 30, 40]. Whether SARS-CoV-2 infection directly impacts the homeostasis of the kidney or is a result of multi-organ failure remains poorly understood; however acute kidney injury (AKI) is commonly reported in patients with severe COVID-19 [48, 93, 94]. Small subsets of COVID-19 patients had signs of renal dysfunction, including proteinuria and hematuria, on hospital admission [48, 93, 95, 96]. Furthermore, nephrologists have observed that a significant number of patients with severe COVID-19 develop AKI, becoming dependent on hemofiltration or hemodialysis [94]. Panel nephrologists noted that renal pathology varies and that most of these patients seem to recover, but with some delay. Physicians should consider that AKI in patients with COVID-19 could be related to being hypovolemic due to critical illness. Moreover, physicians should consider that patients with ATTR amyloidosis might have an increased risk for renal dysfunction if they contract severe COVID-19 and then are managed with relative intravascular volume reduction to maintain oxygenation during ICU care.

### Observations of COVID-19 in patients with ATTR amyloidosis

The current number of patients with ATTR amyloidosis who have acquired COVID-19 is rising and being assessed by physicians worldwide. By May 2019 (during this global medical roundtable), only 6 of the 25 physicians reported clinical experience with patients with ATTR amyloidosis testing positive for COVID-19. In the early months of the global pandemic, physicians from the United States remarked that few patients with ATTR amyloidosis have been infected with COVID-19 likely due to adherence to recommended mitigation strategies (eg, physical distancing and quarantine). In Spain at the beginning of the pandemic, roundtable experts experienced cases in which patients with ATTR amyloidosis had restricted access to ICUs, possibly due to the perception that poor outcomes were inevitable for these patients. This observation is important as hospitals continue to adopt crisis measures as admission capacity and hospital beds, particularly in intensive care, have been in the past and may in the future become saturated. By the end of 2020, most physicians on this panel had experienced lockdown restrictions as well as returns to in-person visits with enhanced cleaning and safety protocols. As the pandemic rises and wanes, there is a need to make other physicians aware that the prognosis for patients with ATTR amyloidosis should be assessed and individualized, and that patients with ATTR amyloidosis should be considered candidates for ICU care if necessary. ATTR amyloidosis is a treatable disease, and the approved effective therapies can slow disease progression, preserve quality of life, and increase the longevity of these patients [97–101].

### The impact of COVID-19 on ATTR amyloidosis diagnosis and treatment

The non-specific, heterogenous, multisystem clinical manifestations of ATTR amyloidosis often make the diagnosis challenging. Early recognition and an elevated index of suspicion through recognition of a constellation of signs and more widespread awareness and testing can lead to earlier diagnosis and treatment [26, 97–101]. Delayed treatment may lead to irretrievable loss of quality of life and significant progression of clinical manifestations, specifically polyneuropathy and cardiomyopathy [26, 97–101].

The COVID-19 pandemic has brought several impediments to the diagnosis and management of patients with ATTR amyloidosis. Diagnostic testing to confirm suspicion of ATTR amyloidosis was initially restricted at many centers because of hospital and clinic closures and a shift in resources toward managing COVID-19. These restrictions have improved at many centers because of increased capacity for testing patients for SARS-CoV-2 infection and improved management of severe COVID-19 disease. Roundtable experts agreed on the need to be flexible and adapt to new ways of working during the pandemic. Globally, many physicians have altered the diagnostic pathway to circumvent challenges relating to patient travel, diagnostic testing, and telemedicine. This has varied by country and local regulations established in response to COVID-19. Telemedicine has limited the ability of physicians to perform examinations and thoroughly diagnose patients using neuropathy assessments, electrodiagnostic testing, and/or obtain skin biopsies for epidermal nerve fiber density. Recently, some clinics have been conducting a limited number of electromyography and skin biopsies, although there is an increased wait time to schedule these procedures in such a way that minimizes the number of patients on site. Specialists agreed that a nerve conduction test is not mandatory to make a diagnosis during the pandemic. If a patient presents with a classic phenotype of ATTR-PN, physicians agreed that a diagnosis can be made without electromyography and nerve conduction studies and diagnosis should not be delayed by waiting for availability of such testing to return. Studies have shown that early treatment of patients with ATTR amyloidosis with effective disease-modifying therapies is critical to maintain a patient’s quality of life and physical function [97–101]. Physicians should be aware that permanent loss of function and quality of life could result for patients who cannot be diagnosed and started on therapy in a timely manner.

Panel experts emphasized that interruption in treatment of ATTR amyloidosis because of COVID-19 should be avoided. At the time of the roundtable (May 2020), most physicians noted that there had been no change in therapy for their current patients with ATTR amyloidosis during the COVID-19 pandemic. As of the writing this article, the aim of treating physicians remains to ensure that the patient has adequate treatment/management of his or her disease with whatever treatment option is most appropriate given the additional challenges of COVID-19. The International Society of Amyloidosis (ISA) recommends that, due to the multisystem nature of ATTR amyloidosis, patients infected by SARS-CoV-2 be considered at increased risk and telemedicine should be employed when possible to minimize travel for treatment and evaluations [102]. In addition, the ISA recommends patients should be enrolled in home care programs when possible to receive treatment [102]. Many panel experts have implemented telemedicine and at home care to manage treatment and assess disease progression of patients with ATTR amyloidosis. Although some aspects of diagnosis and patient management have been impeded, panel physicians have been able to successfully perform the initial workup of de novo patients and maintain continuity of care for existing patients despite closures due to COVID-19 using remotely coordinated multidisciplinary teams. Initial remote evaluations can be accomplished by obtaining appropriate laboratory and diagnostic tests locally and having results available to the consulting physician at the specialty center. Sometimes the evaluation is limited by the availability of specific testing locally (cardiac imaging, for example), thus in-person follow-up visits are recommended when safe from a pandemic perspective. In some countries, telemedicine may be a challenge as only patients with COVID-19 are being seen by physicians in hospitals, and at-home nursing care is not available, which in some areas has had a disastrous impact on patient management, assessment of disease progression, and execution of clinical trials. For example, the pandemic has had a huge impact on access to commercial therapies for ATTR amyloidosis in Latin America, due to the timing of the contract renewal and necessary guideline updates for drugs.

As hospitals in different regions around the world have begun to reopen, physicians have noted that patients are often afraid to come to the hospital, causing challenges in diagnosis, assessment of progression, and treatment. Patients must be aware of the competing risks of delaying diagnosis or interrupting treatment for ATTR amyloidosis and the risk for SARS-CoV-2 infection in the hospital, where everyone who enters the facility is screened for COVID-19. Levels of transmissions in hospitals are now much lower than in the community due to implementation of mitigation strategies such as physical distancing and use of personal protective equipment.

In addition, the pandemic has seriously impacted clinical trial progress. Enrollment in many studies was initially shut down at many of our institutions. Ongoing studies have been modified with remote consent, monitoring, and outcome measures. At many sites, clinical trials have reengaged but still face challenges because of restriction of outside study monitors onsite. As clinical activities reengage, some patients are reluctant to travel and visit hospitals. And an increased risk for exposure to SARS-CoV-2 while traveling is a concern as physicians/investigators try to bring patients to study sites. As clinics reopen with enhanced cleaning and safety measures, protocols must be modified to make patients comfortable enrolling, considering most must travel to the site where the trial is being conducted. Also, sponsors should consider budgeting for repeat COVID-19 testing for active infection, in part to protect participants and study staff.

At the drafting of this article (January 2021), multiple highly effective COVID-19 vaccines have been approved for emergency use in the United States, EU, and elsewhere. While vaccine rollout is expected to continue over the majority of 2021, existing safety measures and restrictions to control the spread of the viral infection will likely be maintained in the immediate future.

## Conclusions

Overall, physicians from all specialties concluded that patients with ATTR amyloidosis who develop COVID-19 have a higher risk of mortality, due to age and other comorbidities, such as hypertension and diabetes, necessitating additional precautions and specialized management (Table 2). More research is needed to fill remaining gaps in knowledge to better understand the clinical impact of COVID-19 on patients with ATTR amyloidosis. Several questions about the clinical manifestation of COVID-19 in patients with ATTR amyloidosis remain to be addressed. Are patients with ATTR amyloidosis more susceptible to poor clinical outcomes due to COVID-19? How might overlapping clinical manifestations have detrimental effects on patients with ATTR amyloidosis who contract COVID-19? COVID-19 can lead to death, often due to multisystem organ failure. How might the multisystem organ effects of ATTR amyloidosis accelerate progression upon developing COVID-19 from a SARS-CoV-2 infection? While waiting for evidence-based recommendations and roll out of emerging vaccines and therapies, preventative measures should be implemented, including physical distancing and precautions during medical visits.

**Table 2:** Physician perspectives on the impact of COVID-19 in patients with ATTR amyloidosis

Key highlights	
•Older patients with ATTR amyloidosis are at increased risk for developing severe COVID-19, requiring social distancing, use of protective masks, and frequent hand washing •Many older patients with ATTR amyloidosis share comorbidities known to increase morbidity and mortality risk in COVID-19 •Patients with cardiac ATTR amyloidosis should be aware of their predisposition to complications if they develop COVID-19, particularly stroke and cardiac-related issues •Differential access to care and the utilization of telehealth may more greatly impact older individuals with ATTR amyloidosis •Laboratory test results, such as elevated cardiac biomarkers, may be seen in both ATTR amyloidosis and COVID-19, confusing interpretation •Limitations of in-person evaluations and performing diagnostic evaluation during the COVID-19 pandemic limits the ability to diagnose and follow progression of patients with ATTR amyloidosis •Because of overwhelmed resources as well as safety of patients and healthcare personal, new approaches to clinical research including remote assessments need to be considered •More research is needed to fill remaining gaps in knowledge to better understand the real-world clinical impact of COVID-19 on patients with ATTR amyloidosis	

*ATTR* Amyloid transthyretin, *COVID-19* Coronavirus disease 2019

## Data Availability

Not applicable.

## Abbreviations

ACE2: Angiotensin converting enzyme 2
ACS: Acute coronary syndromes
AKI: Acute kidney injury
ARDS: Acute respiratory distress syndrome
ATN: Acute tubular necrosis
ATTR: Amyloid transthyretin
ATTRv: Hereditary amyloid transthyretin, v is for variant
ATTRwt: Wild-type amyloid transthyretin
BNP: Brain-type natriuretic peptide
CNS: Central nervous system
COVID-19: Coronavirus disease 2019
ECMO: Extracorporeal membrane oxygenation
ICU: Intensive Care Unit
LV: Left ventricle
NSTE-ACS: Non-ST-elevation acute coronary syndromes
NT-pro BNP: N-terminal proB-type natriuretic peptide
PNS: Peripheral nervous system
SARS-CoV-2: Severe acute respiratory syndrome coronavirus 2
STEMI: ST-elevation myocardial infarction
TTR: Transthyretin
VV-ECMO: Veno-venous ECMO
